# Case report: A novel mutation in *RTEL1* gene in dyskeratosis congenita

**DOI:** 10.3389/fonc.2023.1098876

**Published:** 2023-03-02

**Authors:** Haider Nisar, Memoona Khan, Qamar Un Nisa Chaudhry, Raheel Iftikhar, Tariq Ghafoor

**Affiliations:** ^1^ Adult and Pediatric Transplant Unit, Armed Forces Bone Marrow Transplant Center/National Institute of Bone Marrow Transplant, Rawalpindi, Pakistan; ^2^ Pathology Department and Stem Cell Research Lab, Armed Forces Bone Marrow Transplant Center/National Institute of Bone Marrow Transplant, Rawalpindi, Pakistan

**Keywords:** dyskeratosis congenita, heterozygous, mutation, RTEL1, telomere biology disorders

## Abstract

Dyskeratosis congenita (DKC), also known as Zinsser–Cole–Engman syndrome, is a telomeropathy typically presenting as a triad of leukoplakia, nail dystrophy, and reticular hyperpigmentation. Reported genetic mutations linked to DKC include *DKC1*, *TINF2*, *TERC*, *TERT*, *C16orf57*, *NOLA2*, *NOLA3*, *WRAP53/TCAB1*, and *RTEL1*. Homozygous, compound heterozygous, and heterozygous mutations in *RTEL1* (*RTEL1*, regulator of telomere elongation helicase 1) gene on chromosome 20q13 are known to cause autosomal dominant as well as recessive DKC. Pathogenic variants of *RTEL1* gene in *DKC* patients include c.2288G>T (p. Gly763Val), c.3791G>A (p. Arg1264His), and *RTEL* p. Arg981Trp. We report a novel homozygous variant of *RTEL1*, transcript ID: ENST00000360203.11, exon 24, c.2060C>T (p.Ala687Val), in a patient of DKC presenting with leukoplakia, dystrophic nails, reticulate pigmentation, and positive family history of a similar phenotype. The novel variant, reported as a variant of uncertain significance, may therefore be considered diagnostic for DKC in a Pakistani population.

## 1 Introduction

DKC is an inherited bone marrow failure syndrome due to telomere biology disorder and presents with heterogenous clinical manifestations (1). Spectra of clinical features vary from the classical triad of reticulated pigmentation of skin, nail dystrophy, and oral leukoplakia to life-threatening complications including bone marrow failure, solid tumor malignancies, pulmonary and liver fibrosis, and immunodeficiencies (2, 3).

The diagnosis of DKC in a proband requires a combination of classical clinical features and molecular criteria; this includes either demonstration of shortened telomere length established *via* automated multicolor flow or identification of one of the genes known to cause DKC depending on the pattern of inheritance. Genes responsible for the disorder and reported in the literature include *DKC1*, *TINF2*, *TERC*, *TERT*, *C16orf57*, *NOLA2*, *NOLA3*, *WRAP53/TCAB1*, and *RTEL1* (4). These mutations are encountered in about 80% of patients meeting the diagnostic criteria of DKC*. RTEL1* (regulator of telomere elongation helicase 1) gene prevents the loss of telomere during cell division, playing a pivotal role in telomeric DNA repair and replication. Mutations in *RTEL1* gene result in loss of genomic stability, shortened telomere lengths, and resultantly DKC phenotype. Molecular testing in most centers comprises targeted gene sequencing through either a customized mutigene panel or a comprehensive genomic testing. Severe variants of DKC have been associated with homozygous mutation in *RTEL1* gene and very short telomeres. Similarly heterozygous mutations are commonly encountered in cases of familial pulmonary fibrosis (5). While identification of one of the variants of uncertain significance does not confirm the diagnosis, it does not exclude the disease per se. We, hereby, report a case of a young male patient with characteristic phenotypic abnormalities associated with DKC, a significant family history of similar phenotypic abnormalities, and a homozygous mutation in *RTEL1*, reported as a variant of uncertain significance (VUS).

## 2 Case report

### 2.1 Patient information and clinical findings

Our patient was seen initially by a general physician (GP), in 2015 at the age of 14 years, complaining of failure to gain weight. There was no history of fever, cough, chronic diarrhea, loss of appetite, weight loss, skin rash, or joint pains. On examination, the patient’s weight was below the third centile for his age. Complete blood counts showed mild thrombocytopenia in 2015 (WBC 3.9 × 10^9^/l, absolute neutrophil count 1.95 × 10^9^/l, hemoglobin 13.1 g/dl with MCV 95 Fl, and platelet count of 85 × 10^9^/l). At that time, GP evaluated the patient on lines of metabolic and endocrine dysfunction. All investigations conducted including liver function tests, renal function tests, serology for hepatitis B and C, thyroid profile, and insulin-like growth factor 1 were within normal limits. Based on history of poor weight gain, celiac screen was advised and his anti-tissue transglutaminase IgA levels were 14.8 (U/ml) (negative <12, borderline (6-9), and positive >18). The patient was started on a gluten-free diet. His duodenal mucosal biopsy revealed total villous atrophy suggestive of celiac disease (Marsh stage 3b). (microscopic exam: villous to crypt ratio <1:1, increased intraepithelial lymphocytes, and a subepithelial tissue infiltrate composed of lymphocytes and plasma cells). The patient remained on a gluten-free diet till 2018, but thrombocytopenia persisted and no increment in weight was seen. He continued to visit local physicians and was advised to start hematinic and multivitamins. Due to persistent thrombocytopenia, the patient was referred to our center for further evaluation and management.

### 2.2 Timeline

#### 2.2.1 Diagnostic assessment at AFBMTC/NIBMT

**Figure 1:**
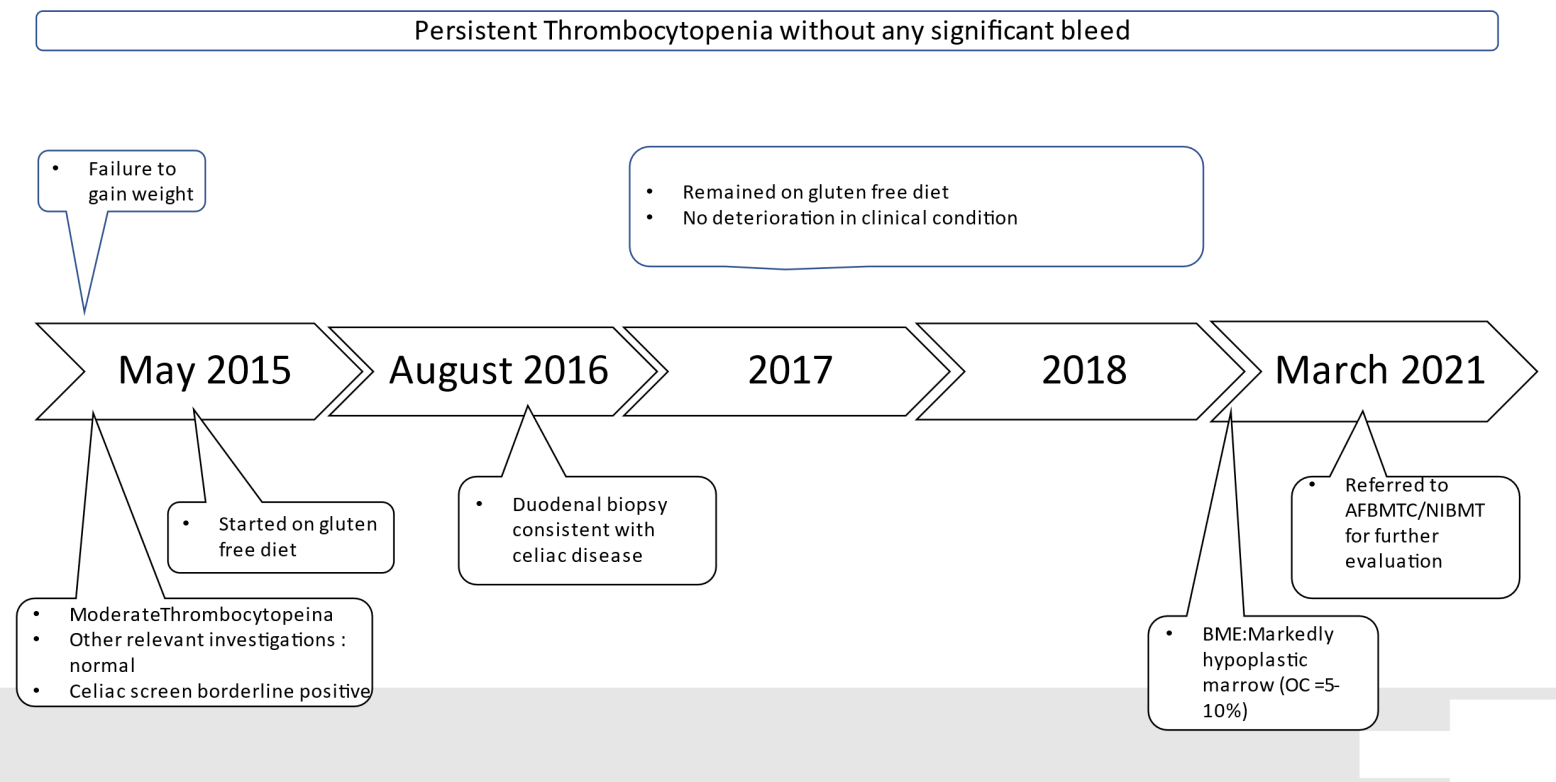
Summary of Clinical events before diagnosis.

**Figure 2:**
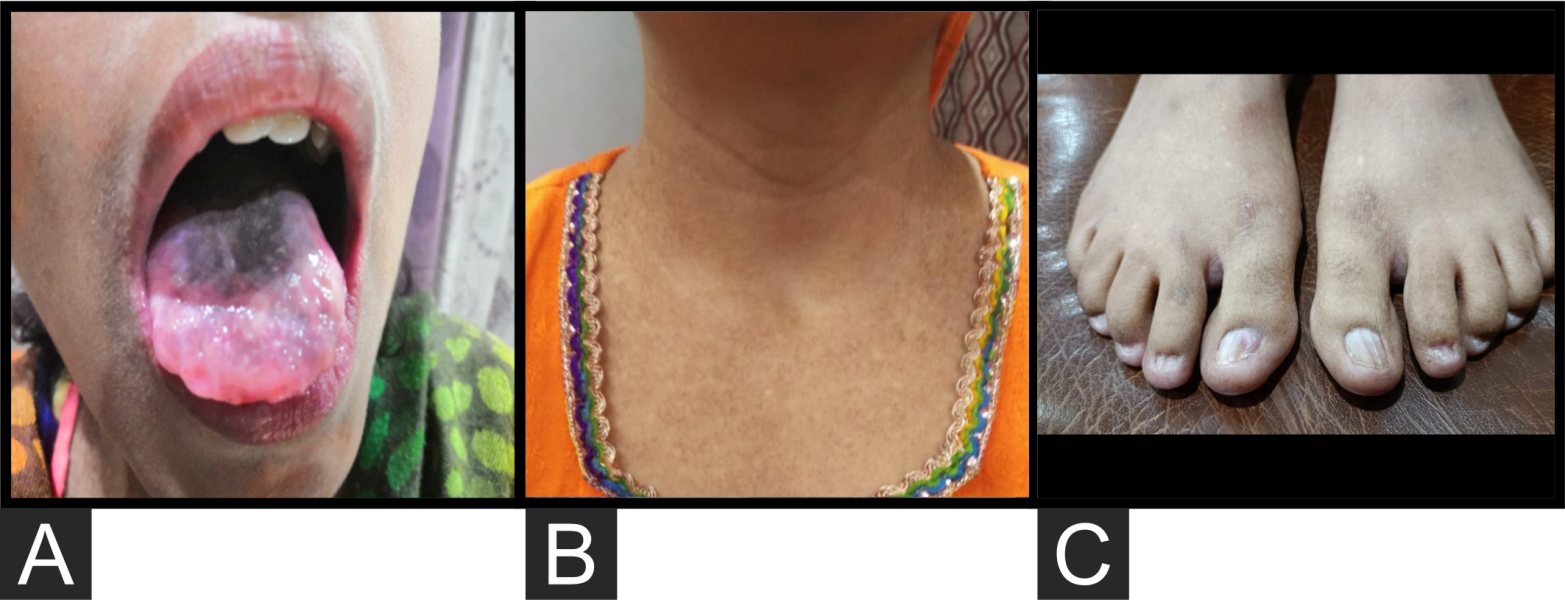
**(A)** Oral lesions. **(B)** Hyperpigmentation over the upper chest. **(C)** Dystrophic nails.

**Figure 3:**
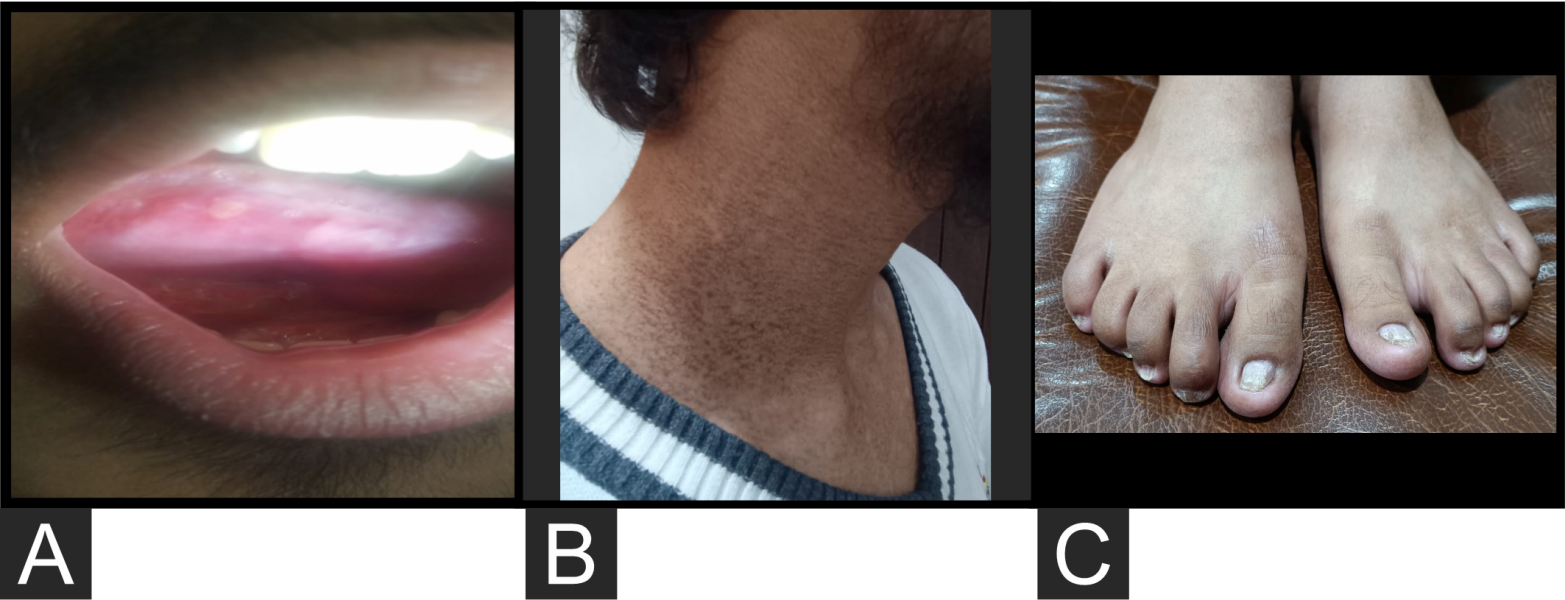
**(A)** Leukoplakia. **(B)** Reticulated hyperpigmentation over the side and front of the neck. **(C)** Dystrophic nails.

The patient presented to our institute in March 2021. A detailed family history revealed that he was born to a consanguineous marriage. He was the youngest of eight siblings with a history of early neonatal death of two siblings and death of one sibling at age of 4.5 years (cause unknown in all). He had an elder sister of 25 years with a history of chronic gastrointestinal (GI) complaints, rash, oral lesions, and nail abnormalities (**Figures 1**, **2**) However, the blood counts of the sister were in normal limits and there was no history of bleeding or infections. All other family members were phenotypically normal. The patient had no significant surgical history. Psychosocial history was unremarkable. On examination, he was active, alert, thin built, and had a weight of 38.8 kg and height 157 cm, both of which were below the third centile for his age. Moreover, he had reticulated hyperpigmentation over the neck and upper chest, dystrophic nails, and leukoplakia (**Figure 3**). His bone marrow examination showed a markedly hypocellular marrow for age with depressed trilineage hematopoiesis, absence of reticulin fibrosis, and infiltration. Cytogenetics revealed a 46 XY male karyotype. Based on family history, classical clinical features, and hypocellular marrow, a provisional diagnosis of inherited marrow failure syndrome most likely dyskeratosis congenita (DKC) was made and the patient was advised genetic testing for confirmation of diagnosis. Because of non-availability of next-generation sequencing in our country, samples of the patient were submitted to Invitae, a clinical diagnostic lab in San Francisco, California, United States, offering DNA-based testing of genetic disorders. Genomic DNA was obtained from the submitted sample, and targeted sequences for nine genes (*CTC1*, *DKC1*, *NHP2*, *NOP10*, *PARN*, *RTEL1*, *TERC*, *TERT*, and *TINF2*) were done. Genomic DNA was enriched for targeted regions using a hybridization-based protocol and sequenced using Illumina technology. All targeted regions were sequenced with a ≥50× depth. Reads were aligned to a reference sequence (GRCh37), and sequence changes were identified and interpreted in the context of a single clinically relevant transcript. Enrichment and analysis focused on the coding sequence of the indicated transcripts, 20 bp of flanking intronic sequence, and other specific genomic regions demonstrated to be causative of disease at the time of assay design. Promoters, untranslated regions, and other non-coding regions were not otherwise interrogated. Exonic deletions and duplications were called using an in-house algorithm that determined the copy number at each target by comparing the read depth for each target in the proband sequence with both mean read depth and read-depth distribution, obtained from a set of clinical samples. Markers across the X and Y chromosomes were analyzed for quality control purposes. Confirmation of presence of location of reportable variants was performed based on stringent criteria established by Invitae, as needed, using one of several validated orthogonal approaches (6). Interpretation reference ranges: benign (normal range): <25 repeat units, uncertain: 25–30 repeat units, pathogenic (full mutation): ≥31 repeat units. A homozygous variant of uncertain significance, *c.2060C>T* (p. Ala687Val) was identified in *RTEL1* gene. There were no alternate candidate variants reported in any of the nine genes investigated for segregation in the pedigree. Keeping in view the positive family history and characteristic triad of leukoplakia, nail dystrophy, and skin pigmentation in the elder sister, a family screening through molecular testing was planned. Samples of the elder brother, sister, and mother were sent to Invitae for genetic testing. Interestingly, the sister was also found to be homozygous for a similar mutation, c.2060C>T in *RTEL1* gene. Moreover, the elder brother and mother’s samples also revealed a heterozygous variant of uncertain significance, *c.2060C>T* (p. Ala687Val) in *RTEL1* gene. To obtain further supporting information regarding pathogenicity of the variant, relative telomere lengths of the proband, his sister, brother, and mother were carried out through qPCR using Richard M. Cawthon 2002 protocol (7). The proband (homozygous for the mutation) and his mother (heterozygous) were found to have a shortened telomere to single-copy gene ratio, whereas the brother and sister had normal ratios. The variant c.2060C>T; A687V was predicted to be conserved missense mutation by Mutation Taster, PhyloP (3.8), and PhastCons (1). This variant could not change the polarity of amino acids as both alanine and valine are neutral and aliphatic amino acids. Functional conservation was also predicted from the physiochemical properties of the variant c.2060C>T, predicted by the ProtParam tool, as shown in the table (**Table 1**; **Supplementary File**). Hence, the variant c.2060C>T could locally affect the protein structure whereas protein features would be conserved. However, RNA sequencing of the variant with functional assay of the protein could not be done because of financial/resource constraints.

**Table 1:** Comparison of physiochemical properties of variant c.2060C>T; A687V.

Properties	Alanine-wild	Valine-mutant
Molecular weight	142366.65	142394.70
Formula	C6272H9875N1827O1864S52	C6274H9879N1827O1864S52
Estimated half-life (mammalian reticulocytes, *in vitro*)	30 h	30 h
Instability index	53.79 (unstable)	53.73 (unstable)
Aliphatic index	74.80	74.95
Grand average of hydropathicity	-0.411	-0.409

### 2.3 Family tree 

#### 2.3.1 Therapeutic intervention and follow-up

**Figure 4:**
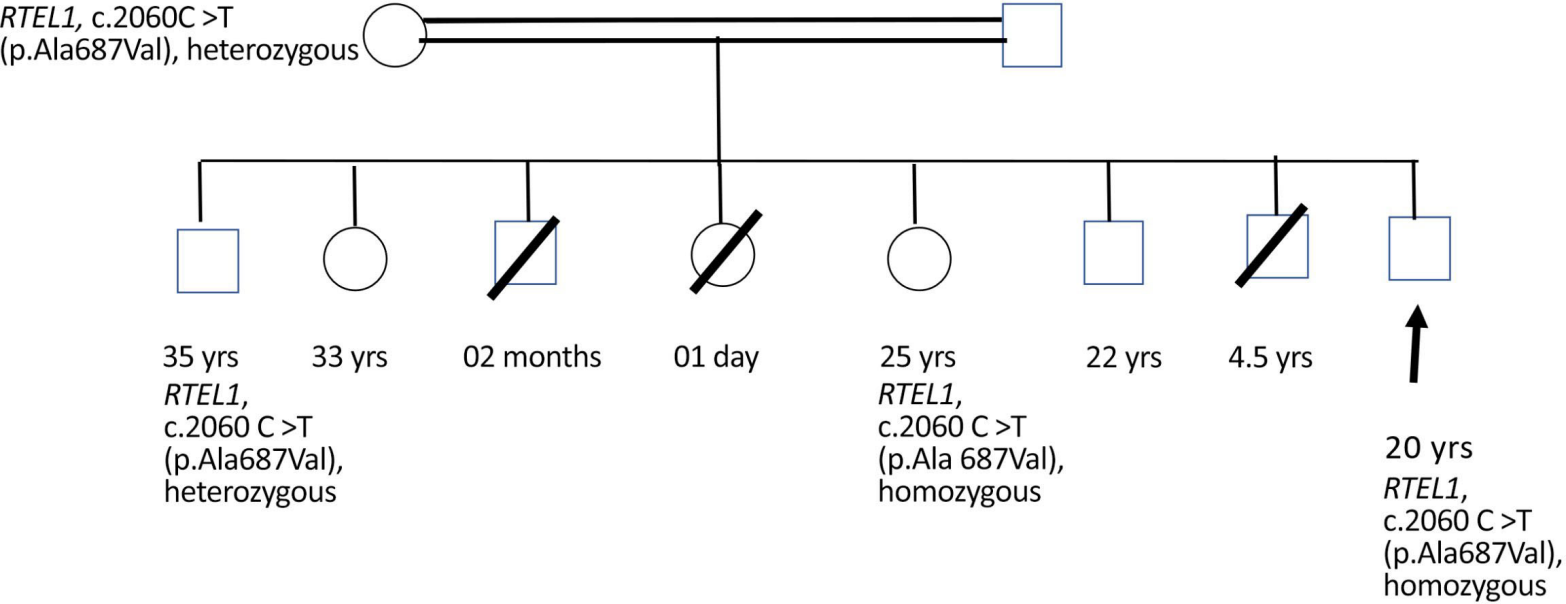
Male sibling: death at 2 months of age, cause not established. Female sibling: death at 24 h of life, cause not established. Male sibling: death at 4.5 years of age, cause not established. 33-year-old female sibling: alive and healthy. 22-year-old male sibling: alive and healthy.

Please refer to **Figure 4** for details of family tree.

The patient was started on oxymetholone (100 mg once a day) along with a tablet of folic acid (5 mg once a day). The patient took the treatment for 6 months (January 2022 to June 2022). His platelet counts remained stable (60,000–70,000/µl), and he has remained transfusion independent. On the last assessment in September 2022, his counts were WBC 3.5 × 10^9^/l, absolute neutrophil count 1.8 × 10^9^/l, hemoglobin 13.4 g/dl with MCV 95 fl, and platelet count of 70 × 10^9^/l. The patient is followed up every 4 weekly currently. At present, the plan is to continue oxymetholone and pursue with a non-transplant strategy as the patient has stable blood counts.

## 3 Discussion

At least 13 types of inherited bone marrow failure syndromes (IBMFS) have been reported in literature so far. DKC, a telomere biology disorder, is an IBMFS, resulting from defect in telomere maintenance. The disease is characterized by a variety of somatic abnormalities with some cases presenting with a classical triad of abnormal skin pigmentation, nail dystrophy, and mucosal leucoplakia (8–11). Several genes have been linked to the pathophysiology of DKC including dyskeratosis congenita 1 (*DKC1*), CTS telomere maintenance complex component 1 (*CTC1*), regulator of telomere elongation helicase 1 (*RTEL1*), TERF 1-interacting nuclear factor 2 (*TINF2*), telomerase RNA component (*TERC*), telomerase reverse transcriptase (*TERT*), adrenocortical dysplasia homolog (*ACD*), NHP2 ribonucleoprotein (*NHP2*), NOP 10 ribonucleoprotein (*NOP10*), poly(A)-specific ribonuclease (*PARN*), nuclear assembly factor 1 (*NAF1*), and WD repeat containing antisense to TP53 (*TCAB1*) (12–14).

*RTEL1* has a vital role in genomic stability through regulation of homologous recombination (HR). The gene is critical for mitotic and meiotic double-strand DNA repair and mutations resulting in enhanced susceptibility to chromosomal breakage (15–17). *RTEL1* deficiency, despite being reported as a major genetic contributor, has not been extensively studied, and the specific clinical outcome with correlation to molecular landscape of the mutation is still under research.

Here, we report a case of DKC presenting with a classical DKC triad and a homozygous variant of *RTEL1* c.2060C>T (p. Ala687Val). This sequence change replaces alanine, which is neutral and non-polar, with valine, which is neutral and non-polar, at codon 687 of the *RTEL1* protein. The variant was reported to be of uncertain clinical significance according to the American College of Medical Genetics and Genomics (ACMG) 2015. At the time this patient was tested, Invitae was using AlamutSplice for these splice predictors. Alamut uses three algorithms for these predictions, MaxEntScan (MES), SpliceSiteFinder-like (SSF-L), and NNSPLICE. At least two of the three scores must be significant for the mutant vs. the wild type to predict a gain of a new acceptor/donor splice site. A score is deemed significant if MES >0, SSF >70, and NNSPLICE >0.4. However, Splice AI did not predict the mutation to affect RNA splicing. Currently, it fulfills the moderate evidence of pathogenicity, PM2, supporting evidence of pathogenicity, PP1, and PP4 and hence does not meet criteria for pathogenic or likely pathogenic (18). Based on expanding ACMG variant classification guidelines into a general framework 2022 based on a model of chronic pancreatitis (19), the variant may even be considered to be classified as disease predisposing based on the clinical history, family screening, zygosity determination, and relative telomere length estimation. We searched the literature describing cases of DKC with *RTEL1* mutations. Speckmann et al, in 2017, described six mutations of *RTEL1* with five being novel biallelic p.Trp456Cys, p.Ile425Thr, p.Cys1244ProfsX17, p.Pro884_Gln885ins53X13, and one with novel heterozygous mutation p.Val796AlafsX4 (20). All six patients had BMF with hypocellular marrows, whereas the classical triad was not reported in any patient. Clinical features of three of these patients were consistent with Hoyeraal-Hreidarsson syndrome. Immunophenotyping of these patients showed immunodeficiency and they suffered from cerebellar hypoplasia and enteropathy. All patients had very short telomere lengths below the first percentile. However, this genomic stability could only be demonstrated in fibroblasts, not the hematopoietic cells. Heterozygous carrier parents of four patients also exhibited significant telomere shortening when subjected to replicative stress (20). Dokal and colleagues had also reported genomic instability demonstrated in fibroblasts of DC patients (21).

According to reported literature, heterozygous *RTEL1*, despite causing telomere shortening, may not manifest phenotypically or cause disease pathology because of the possible compensation by functional wild-type alleles. The same might be the plausible reason behind the molecular analysis of the patient’s mother. Missense heterozygous mutations in *RTEL1* associated with DKC phenotype have recently been reported by Speckmann et al. (20) and also by Ballew et al. (22). Previously, pulmonary fibrosis without hematologic or immunological abnormalities was seen in cases of isolated heterozygous *RTEL1* mutations (23–25).

Despite having a markedly hypocellular marrow, the patient has maintained a fairly stable ANC (lowest 1.32 × 10^9^/l) and Hb (12.3 g/dl) with moderate thrombocytopenia (average platelet count ~ 60,000 to 70,000/µl, lowest platelet counts 43,000/µl) and certainly is not a candidate for HSCT at the moment. We plan to keep him on vigilant monitoring through follow-up by periodic complete blood counts and assessment of clinical examination for development of any complications. We would also need to find a potential donor for the patient in case a need for transplant arises. His elder brother, 36 years of age, despite being completely HLA matched with him, is heterozygous for *RTEL1* mutation. HSCT from a clinically silent heterozygous family member is not recommended in DKC (26, 27).

Our case reports that homozygous *RTEL1* gene variant c.2060C>T (p.Ala687Val), can present with the classical DKC triad.

However, the c.2060C>T variant has NOT been reported in either the gnomAD or ClinVar database to date despite having phenotypic abnormalities in the Pakistani population as reported here. A general representation of South Asians toward the reporting of variant interpretation in genetic diseases is relatively poor compared with other populations with more extensive studies including those with European and American ancestries. Discrepancies in screening efficiency and lack of inclusion of South Asian variants in testing panels are an extension of the issue being expressed here and need to be addressed by increasing the pool of South Asian individuals.

## Data availability statement

The original contributions presented in the study are included in the article/supplementary material. Further inquiries can be directed to the corresponding author.

## Ethics statement

The studies involving human participants were reviewed and approved by Institutional Review Board Committee Armed Forces Bone Marrow Transplant Center. The patients/participants provided their written informed consent to participate in this study. Written informed consent was obtained from the individual(s) for the publication of any potentially identifiable images or data included in this article.

## Author contributions

HN and MK conceived and designed the study and drafted the manuscript. RI critically revised the manuscript. QC also contributed to the revision of the manuscript. All authors contributed to the article and approved the submitted version.
