# Supplementary material for: Case report: A novel mutation in RTEL1 gene in dyskeratosis congenita
Source: Front Oncol. 2023 Mar 2;13:1098876. doi: 10.3389/fonc.2023.1098876 (PMC10017992; doi:10.3389/fonc.2023.1098876)
Supplement: Supplementary file 1 [file DataSheet_1.pdf]

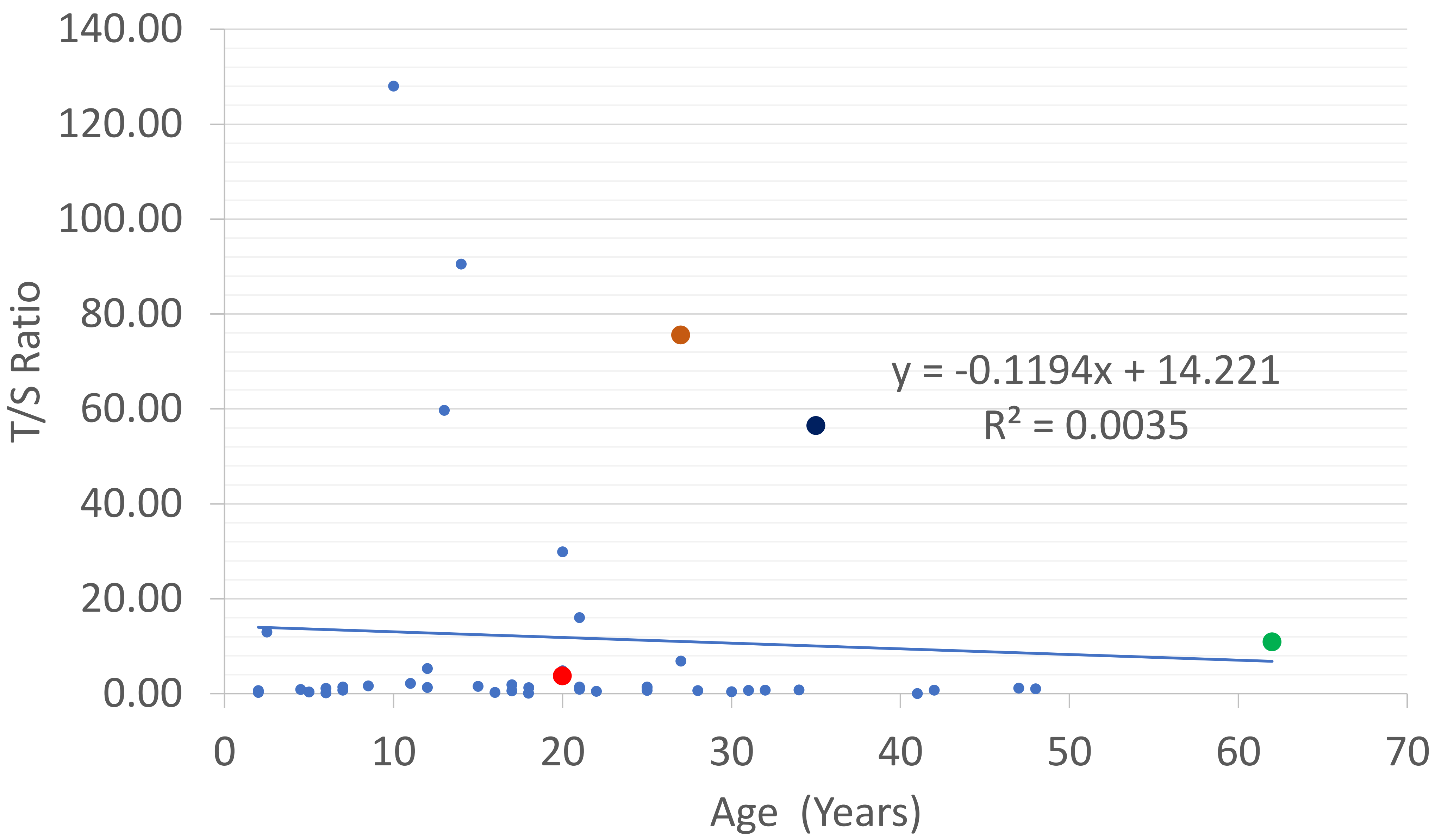

The telomere length (T/S ratio) of patient (Red circle), mother (Green circle), brother (dark blue) and sister (orange) plotted against telomere lengths of normal healthy individuals ages between 2-48 years, using 36B4 as single copy gene (Cawthon, R. M. (2002). Telomere measurement by quantitative PCR. Nucleic Acids Research, 30(10). https://doi.org/10.1093/nar/30.10.e47).

We are already in process of optimizing multiplex Real time PCR using human beta globulin as house keeping gene (Cawthon, R. M. (2009). Telomere length measurement by a novel monochrome multiplex quantitative PCR method. Nucleic Acids Research, 37(3). https://doi.org/10.1093/nar/gkn1027).
